# Steam Explosion Conditions Highly Influence the Biogas Yield of Rice Straw

**DOI:** 10.3390/molecules24193492

**Published:** 2019-09-26

**Authors:** David Steinbach, Dominik Wüst, Simon Zielonka, Johannes Krümpel, Simon Munder, Matthias Pagel, Andrea Kruse

**Affiliations:** 1 Institute of Agricultural Engineering, University of Hohenheim, Garbenstrasse 9, 70599 Stuttgart, Germany; wuest.dominik@uni-hohenheim.de (D.W.); S_Munder@uni-hohenheim.de (S.M.); Andrea_Kruse@uni-hohenheim.de (A.K.); 2 Karlsruhe Institute of Technology (KIT), Institute for Catalysis Research and Technology, Hermann-von-Helmholtz-Platz 1, 76344 Eggenstein-Leopoldshafen, Germany; matthias.pagel@kit.edu; 3 State Institute of Agricultural Engineering and Bioenergy, University of Hohenheim, Garbenstrasse 9, 70599 Stuttgart, Germany; simon.zielonka@uni-hohenheim.de (S.Z.); j.kruempel@uni-hohenheim.de (J.K.)

**Keywords:** steam treatment, pretreatment, lignocellulose, anaerobic digestion, biochemical methane potential, biomethane

## Abstract

Straws are agricultural residues that can be used to produce biomethane by anaerobic digestion. The methane yield of rice straw is lower than other straws. Steam explosion was investigated as a pretreatment to increase methane production. Pretreatment conditions with varying reaction times (12–30 min) and maximum temperatures (162–240 °C) were applied. The pretreated material was characterized for its composition and thermal and morphological properties. When the steam explosion was performed with a moderate severity parameter of *S_0_* = 4.1 min, the methane yield was increased by 32% compared to untreated rice straw. This study shows that a harsher pretreatment at *S_0_* > 4.3 min causes a drastic reduction of methane yield because inert condensation products are formed from hemicelluloses.

## 1. Introduction

Rice straw is one of the most abundant lignocellulosic agricultural residues worldwide and is produced mostly in Asia as a byproduct of rice production. Rice was in the third place of crop production in 2013 with a world annual production of 746 million tons [1]. The production of rice straw as a byproduct can be estimated at about 1120 million tons using a straw-to-grain ratio of 1.5 [2]. A part of this agricultural residue is used, for example, as cattle feed [3]. Unfortunately, open-field burning of straw, which increases air pollution, is a common practice in Asia [4,5,6].

Possible energetic utilization of rice straw is limited by its low bulk density, which makes large-scale, centralized conversion technologies uneconomical. Therefore, decentralized conversion routs are of special interest. One such method is anaerobic digestion of lignocellulosic biomass, which is a most efficient conversion technology regarding the energy output-to-input ratio [7,8,9].

However, the structure of lignocellulosic biomass generally causes a low digestibility during anaerobic digestion because lignocellulose is a strongly connected composite of cellulose, hemicelluloses, and lignin. Cellulose is a linear polymer consisting of glucose building blocks linked together by glycosidic bonds. The intermolecular hydrogen bonds between adjacent cellulose chains result in a highly ordered, water-insoluble configuration that makes cellulose crystalline [10]. Hemicelluloses are a group of amorphous heteropolymers that are significantly shorter than cellulose macromolecules. They are fixed in the lignocellulosic fiber structure because they provide a linkage between lignin and cellulose. Hemicelluloses are connected to lignin via covalent links and surround cellulose. Lignin is a complex, three-dimensional macromolecule constructed of phenylpropane units and is non-biodegradable via anaerobic digestion [8].

Hydrolysis is the rate-limiting step in the biogas process when a solid feedstock, such as lignocellulose, is used [8]. A pretreatment is required to increase the availability of cellulose and hemicellulose for the hydrolysis step. Pretreatment generally aims at the disintegration and separation of biomass to release the different components [11]. A variety of pretreatment methods have been under investigation for enhancing the biogas production of lignocellulose [9,12,13]. Among these methods, steam pretreatment was rated with a high potential [12]. It is a relatively inexpensive pretreatment as it does not require the addition of an external catalyst [14]. Also, it has energetic advantages because it can be carried out with approximately 1.5 kg of steam per kilogram of biomass, compared to 5–10 kg of hot water usually required for liquid phase pretreatments [15].

Steam explosion converts biomass at elevated pressures and temperatures in a steam atmosphere, followed by mechanical disruption of the biomass by discharging to atmospheric pressure. Steam hydrolysis is similar to the steam explosion process, except that it avoids the discharge of rapid pressure. Temperatures ranging from 140–240 °C have been applied over a wide range of residence times [16]. Ferreira et al. [17] named an optimum for steam explosion conditions of 150–220 °C and 5–20 min. They pointed out that pretreatment conditions that are too severe are unfavorable due to the formation of phenolic and heterocyclic compounds (e.g., furfural, hydroxymethylfurfural (HMF), and soluble phenolic compounds). These compounds could inhibit methane production, but the methane-producing microorganisms are, however, capable of adapting to such compounds at smaller concentrations [12].

Thermally labile acetyl groups in hemicelluloses are cleaved during steam explosion, and acetic acid is formed [11]. The liberated organic acids catalyze hydrolysis reactions of hemicelluloses and degradation reactions of a small part of lignin. The residue after steam explosion consists of cellulose, a chemically modified lignin, and residual hemicelluloses. The sum of hemicelluloses in the residue, as well as dissolved hemicellulose-derived sugars, declines with the severity of the pretreatment because of (1) furfural formation by dehydration of pentoses and (2) secondary reactions of dissolved compounds leading to solid pseudolignin by condensation reactions [18].

Steam explosion has been performed with various kinds of lignocellulosic biomasses prior to anaerobic digestion including triticale [19], corn [20], wheat straw [17,21,22,23,24,25,26], hay [27], sugarcane bagasse [28,29], sugarcane straw [29], rape straw [30], bulrush [31,32], miscanthus [33], birch [34], willow [35,36], and cedar [37]. Steam explosion of rice straw for anaerobic digestion was investigated in the study of Zhou et al. [38] who performed steam explosion under narrower parameter conditions (200–220 °C, 1–4 min) and focused on the microbial communities during anaerobic digestion. Other studies have focused on steam explosion of rice straw as pretreatment for enzymatic hydrolysis to obtain fermentable sugars [39,40,41].

The steam explosion pretreatment generally increases the specific methane yields while also increasing the speed of anaerobic degradation. However, the reported increase in methane yield ranged from 0%–5% [22,23] to 10%–30% [17,19,20,21,24,26,27,28,29,30,32] and up to 50%–345% [29,33,34,36,37,38] because of the differences in (1) steam explosion reactor setups and reaction conditions, (2) lignocellulosic plants species and (3) digestion procedures.

The aim of this work was to evaluate the influence of a previous steam explosion in a batch system on the anaerobic digestion of rice straw. Therefore, the steam explosion of rice straw was performed at different severities. The pretreated material was chemically characterized and, thereafter, subjected to batch anaerobic digestion tests to determine the methane yield.

## 2. Materials and Methods

### 2.1. Plant Material

Field-dried rice straw of the *Bahia* variety (*Oryza sativa* var. *Bahia*) was acquired from the Ebro Delta, Spain. The straw was cut in a Viking GE260 chaff cutter (Viking GmbH, Kufstein, Austria) to a length of less than 100 mm. Fines were removed by manual sifting using a perforated sieve with a hole diameter of 3.9 mm. The composition of the plant material is shown in Table 1. Elemental composition and ash content were determined according to the procedures listed in Section 2.3. Fiber analysis was performed via the Van Soest method.

### 2.2. Steam Explosion Pretreatment

**Figure 1:**
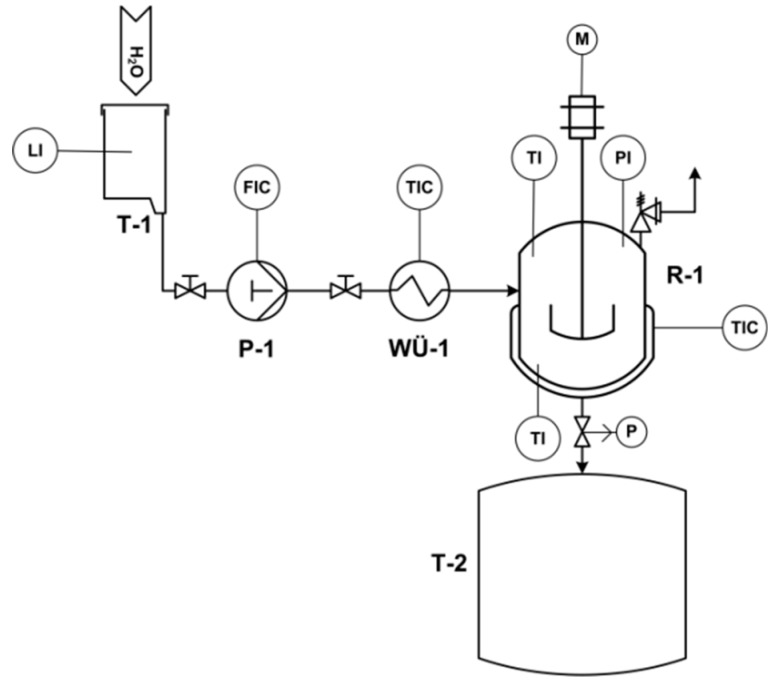
P&ID of the steam explosion miniplant. T-1: deionized water tank, P-1: HPLC pump, WÜ-1: electrical preheater for steam generation, R-1: steam explosion reactor with surrounding heating tape and insulation, and T-2: flash tank.

The steam explosion was performed in a miniplant designed and constructed at the Karlsruhe Institute of Technology. The reaction vessel of the miniplant consisted of a stainless-steel reactor with a volume of 1 L and was constantly agitated with a cross-arm stirrer at 8 min^−1^. Before each experiment, 26.8 ± 1.2 g of straw was inserted into the reactor. The reactor was electrically heated to a temperature of 110 °C. A constant flow of 5 g min^−1^ of steam was then introduced into the reactor. The explosion is technically a rapid equalization of pressure performed by pneumatically opening a ball valve. Volatile products and steam were discharged by the explosion step into a 240 L flash tank. The pretreated rice straw remained in the reactor and was manually removed, and stored at 4 °C. Figure 1 provides a piping and instrumentation diagram (P&ID) of the miniplant.

The reaction temperature was monitored by two type K thermocouples installed in the top and the bottom of the reactor. The arithmetic mean of the two temperatures measured was used for the calculation of the severity parameter *S_0_*, combining time *t* and temperature *T* of the steam pretreatment in a single factor [18]. Equation 1 describes the time integral of *S_0_*, which was used to describe the non-isothermal character of the heating process [42].

(1) $$S_{0}=log\;\overset{t}{\underset{0}{\int }}exp\left(\frac{T\;\left[{}^{\circ}C\right]-100\;{}^{\circ}C}{14.75}\right)dt$$

Different pretreatment conditions with varying steam input times (12–30 min), steam input masses (60–150 g), and maximum temperatures (162–240 °C) were tested. Table 2 provides an overview of the experimental setup. The temperature profile during heat up is shown in the Supplementary Materials.

### 2.3. Chemical Analysis of Untreated Rice Straw and Steam-Exploded Residue

The dry matter content of the untreated material was determined in triplicate by drying the rice straw at 105 °C for 16 h based on DIN EN 14774-1. The steam-exploded residue was dried at 40 °C for 87 h until its weight balanced to reduce evaporation of easily volatile reaction products of the steam explosion. The ash content of the untreated material was determined in triplicate via incineration in an electrically heated muffle oven at 550 °C for 4 h based on DIN EN 14775. The ash content of steam-exploded residues was measured in single runs. The organic dry matter content, which is also called volatile solids, was calculated from the difference between dry matter and ash content.

The stoichiometric carbon, hydrogen, nitrogen, and sulfur content (CHNS) was analyzed chromatically in a Vario EL cube (Elementar Analysesysteme GmbH, Langenselbold, Germany). The milled and dried samples of untreated straw and steam-exploded residue were analyzed in triplicate for CHNS. Sample milling to powder was performed in a freezer mill. The oxygen content of rice straw was estimated, closing the gap between CHNS and ash content to 100 wt.%. Other elements in the rice straw were analyzed after quantitative dissolution (HNO_3_, HCl, HF 6:2:1 v v^−1^) by ICP-OES type 725 (Agilent, Santa Clara, CA, USA).

Thermogravimetric analysis was performed with milled and dried samples in single runs at a STA 449 F5 (Netzsch-Gerätebau GmbH, Selb, Germany) with a heating rate of 10 K min^−1^ and nitrogen as the inerting agent. The thermogram obtained was differentiated to obtain the differential thermogravimetry curve (DTG).

The surface structures of dried untreated straw and steam-exploded residue were investigated via scanning electron microscopy (SEM) in a LEO 982 Gemini (Carl Zeiss AG, Jena, Germany) equipped with a Schottky-type thermal field emission cathode, secondary electron detectors (Everhart-Thornley, inlens), and a backscattered electron detector.

The acid-insoluble lignin content (Klason lignin) and acid-insoluble ash content were determined via an “ASTM protocol” [43] in triplicate for the untreated straw and in single runs for the steam-exploded residues.

Water-soluble components of rice straw and steam-exploded residues were extracted with hot water. Therefore, about 0.9 g milled and dried sample was introduced in an extraction thimble, which was placed in a boiling water bath for 3 h. Water extraction was performed in triplicate for untreated straw and in single runs for steam-exploded residues. After extraction, the liquid was analyzed via HPLC (Deutsche Metrohm GmbH, Filderstadt, Germany). Glucose and xylose were separated via HPLC at 35 °C in a Metrosep Carb 2 column (Deutsche Metrohm GmbH, Filderstadt, Germany) and quantified by an amperometric detector. An eluent with 0.1 mol L^−1^ sodium hydroxide and 0.01 mol L^−1^ sodium acetate was used with a flow rate of 0.5 mL min^−1^. Hydroxymethylfurfural (HMF) and furfural were separated via HPLC at 20 °C using a Lichrospher 100 RP-18 column (Merck KGaA, Darmstadt, Germany) and quantified by a UV detector at 290 nm. A water–acetonitrile eluent (9:1 v v^−1^) was used at a flow rate of 1.4 mL min^−1^.

### 2.4. Specific Biogas Yield

The Hohenheim biogas yield test was used to assess the biochemical methane potential (BMP) of untreated and pretreated rice straw. The pretreated rice straw after steam explosion was directly used for the biogas yield test without any water washing. The Hohenheim biogas yield test is a feasible and well-established laboratory batch test developed at the University of Hohenheim and featured in the VDI-Guideline 4630-Digestion of organic materials [44]. It is used to evaluate and compare the methane production of different substrates as well as their biodegradability and gas production kinetics. Glass syringes (100 mL) were used as digesters and for gas storage. The syringes were fitted into a motor-driven rotor for mixing the sample, which was placed inside an incubator. Every run included a control variant and two standard substrates to ensure the correctness of the results and the comparability of different batches [45,46]. The methane percentage was measured by a gas transducer AGM 10 (Pronova Analysetechnik, Berlin, Germany) with a nondispersive infrared (NDIR) sensor, while the amount of produced biogas was recorded with an accuracy of 1 mL. Three replications were performed for each sample. The gas yield is expressed as m^3^_N_ kg _DM_^−1^, corrected for the gas production of the inoculum, and expressed for standard atmosphere (273.15 K, 1013.25 hPa).

### 2.5. Theoretical Methane and Biogas Yield

The theoretical maximum gas production from an organic substrate with the elemental composition C_a_H_b_O_c_N_d_S_e_ can be determined stoichiometrically, see Equation (2) [47].

(2) $$C_{a}H_{b}O_{c}N_{d}S_{e}+\left(a-\frac{b}{4}-\frac{c}{2}+\frac{3d}{4}+\frac{e}{2}\right)\cdot H_{2}O\;\to \;\left(\frac{a}{2}+\frac{b}{8}-\frac{c}{4}-\frac{3d}{8}-\frac{e}{4}\right)\cdot CH_{4}+\;\left(\frac{a}{2}-\frac{b}{8}+\frac{c}{4}+\frac{3d}{8}+\frac{e}{4}\right)\cdot CO_{2}+d\cdot NH_{3}+e\cdot H_{2}S\;.$$

The maximal methane yield (*Y_CH4_*), expressed as norm cubic meters per kilogram of organic dry matter (m^3^_N_ kg _DM_^−1^), can be obtained by Equation (3) using the molar volume of an ideal gas (22.4 mol L^−1^). However, this theoretic value can never be achieved with lignocellulosic biomass in reality because lignocellulose contains compounds that are considered not biodegradable, such as lignin.

(3) $$Y_{CH_{4}}=\;\frac{22.4\cdot \left(\frac{a}{2}+\frac{b}{8}-\frac{c}{4}-\frac{3d}{8}-\frac{e}{4}\right)}{12a+b+16c+14d+32e}.$$

## 3. Results and Discussion

### 3.1. Characterization of the Solid Residue after Steam Explosion

#### 3.1.1. Chemical Composition

**Figure 2:**
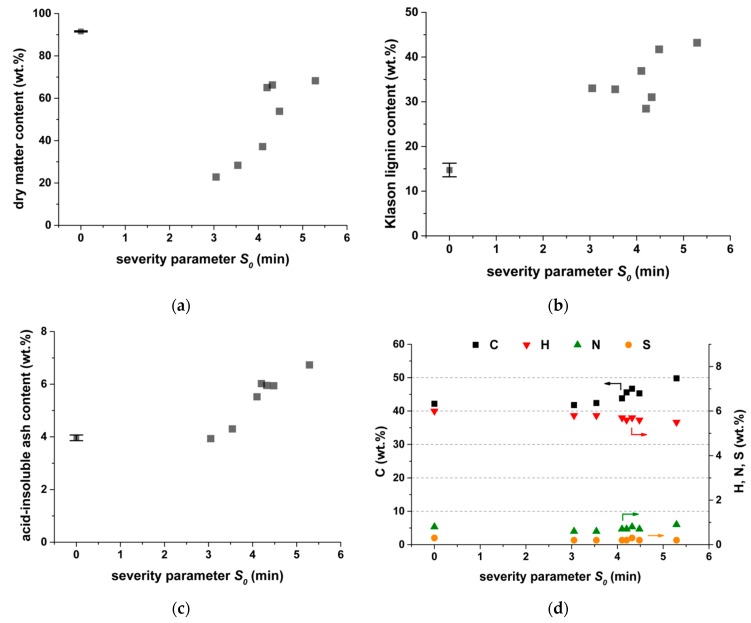
Chemical composition of untreated and pretreated rice straw depending on the severity parameter *S_0_* of the steam explosion, (**a**) dry matter content, (**b**) Klason lignin content, (**c**) acid-insoluble ash content, and (**d**) elemental composition.

The field-dried rice straw had a dry matter content of 91.6 wt.%, which was generally reduced during steam explosion (Figure 2a). However, the dry matter content among the residues increased with severity. An explanation might be that the residue becomes more hydrophobic at higher reaction severity, because of a decrease in the number of polar functional groups. The mass of steam introduced into the reactor might also influence the dry matter content. The experiments with the lowest steam inputs, namely *S_0_* = 4.20, 4.32 and 5.29 min, yielded the highest dry matter contents of 65, 66 and 68 wt.%, respectively.

The acid-insoluble lignin of water-free rice straw accounted for 14.7 wt.% and increased to 28–42 wt.% after steam explosion (Figure 2b). This relative increase has two causes: (1) hemicelluloses, and partly cellulose, are converted during steam explosion to water-soluble or volatile compounds. Thereby, the content of the more inert lignin is increased in the solid residue. (2) Hemicelluloses decompose and repolymerize to form a more stable solid that cannot be hydrolyzed to water-soluble components during the Klason method [11]. These repolymerization or condensation reactions involve not only hemicellulose-derived byproducts but also lignin [16]. These repolymerized pseudolignin compounds are more stable and increase the acid-insoluble lignin content.

The acid-insoluble ash content at low severity parameters was similar to the untreated material and accounts for 4.0 wt.%. The ash content increased at high severity (Figure 2c). This can also be attributed to the conversion of hemicelluloses, and partly cellulose, during steam explosion to water-soluble or volatile compounds. Thereby, the content of inert acid-insoluble ash increased in the solid residue. This parameter could also be used to roughly estimate the weight loss of organic material during steam explosion. This weight loss of organic material should be considered when the cascading of steam explosion and biogas production is evaluated.

Figure 2d shows the elemental composition of solid steam-exploded residues. The carbon content in the solid residue rose at high severity. By contrast, the hydrogen content decreased. This can be explained by the removal of hydrogen- and oxygen-rich volatile compounds during the steam explosion; thus, a carbon-rich solid residue remained.

#### 3.1.2. Water-Extractable Components

**Figure 3:**
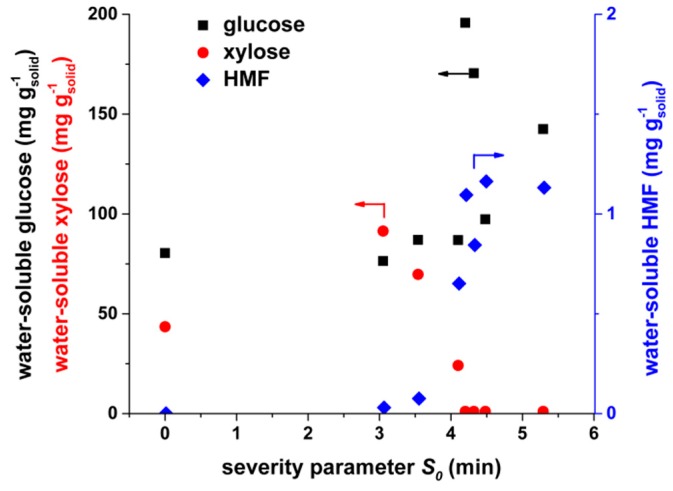
Water-soluble components (glucose, xylose, and hydroxymethylfurfural (HMF)) of untreated rice straw and pretreated residue depending on the severity parameter *S_0_* of the steam explosion. Extraction in boiling water for 3 h.

After the hot water extraction of untreated rice straw, 80 mg g^−1^ glucose and 44 mg g^−1^ xylose were obtained in the liquid phase (Figure 3). Glucose was either present in the rice straw as free monosaccharide or originated from the degradation of polymers like starch or hemicelluloses [48]. In the latter case, the amorphous or rather labile hemicelluloses may partly hydrolyze during water extraction to yield glucose (but also xylose). No HMF was obtained after the water extraction of untreated rice straw; therefore, no dehydration of glucose occurred.

Compared to untreated rice straw, water-extractable xylose increased at a low severity of steam explosion. Xylose was no longer found in the extract at high severities (*S_0_* > 4.1 min). A possible explanation is that hemicelluloses are hydrolyzed to release additional xylose at low severities. At high severities, the released xylose is quickly decomposed to furfural [35]. Water-extractable HMF increases strongly with the severity factor. Thus, hexoses, such as glucose, are dehydrated at a higher severity to form HMF. The HMF is known as a fermentation inhibitor for anaerobic digestion [49]. However, reported values for inhibiting concentrations by HMF seem to be high, usually greater than 5 g L^−1^. Moreover, the intensity of the inhibition is affected by the operational conditions and design of the anaerobic system, as well as the presence of other inhibitors, such as furfural or phenols [49,50]. Both inhibit the activity of acetate in utilizing methanogens.

Furfural was not detected after water-extraction of pretreated material. However, it was present in the gas phase of the steam-explosion reactor (data not shown). A sample of the gas phase was obtained via a relief valve, condensed afterwards, and analyzed for furfural. So, formed furfural was mainly removed as a volatile during steam explosion. A small part of furfural may still be present on the surface of the steam-exploded rice straw subjected to anaerobic digestion.

#### 3.1.3. Thermogravimetric Analysis

The main components of lignocellulosic biomass differ in thermal stability. Hemicelluloses and cellulose consist of glycosidic linkages, which decompose within a narrow temperature region. The amorphous hemicelluloses decomposed at a lower temperature compared to the crystalline cellulose [51,52]. Lignin consists of phenylpropanoid units interconnected by different chemical linkages that have different binding energies [53]. Therefore, the decomposition of lignin proceeded over a broad temperature range.

**Figure 4:**
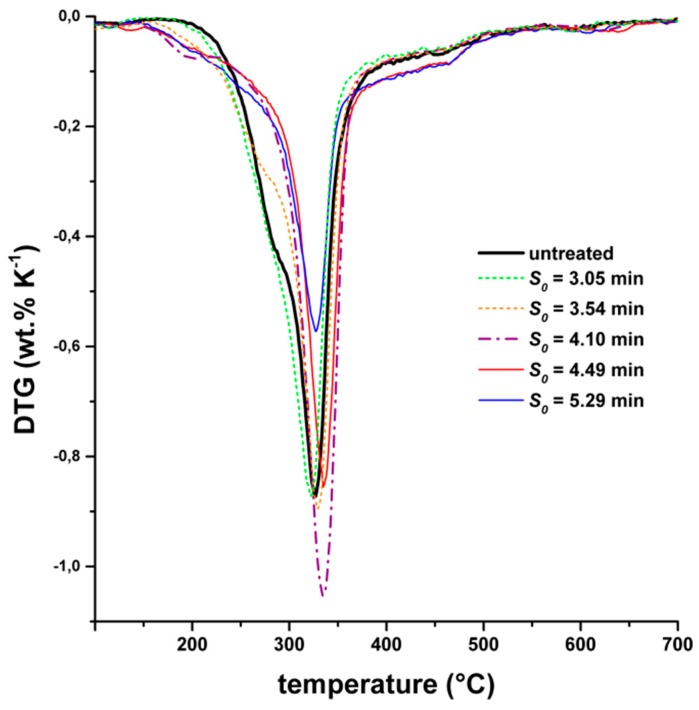
Differential thermogravimetric analysis (DTG) of untreated and pretreated rice straw depending on the severity parameter *S_0_* of the steam explosion.

Figure 4 shows the DTG curves of rice straw. The untreated material showed a peak at around 320 °C, which corresponds to cellulose. Hemicellulose showed a shoulder at 280–300 °C, which is still present for the lowest-severity experiments *S_0_* = 3.05 min and *S_0_* = 3.54 min. At a higher severity, the hemicellulose shoulder disappeared completely, indicating a destruction or alteration of hemicelluloses. An increased mass loss at 380–480 °C was detected for the high-severity experiments (*S_0_* = 4.49 min and *S_0_* = 5.29 min), which could be assigned to more temperature-stable repolymerization products of hemicellulose.

#### 3.1.4. Particle Morphology

**Figure 5:**
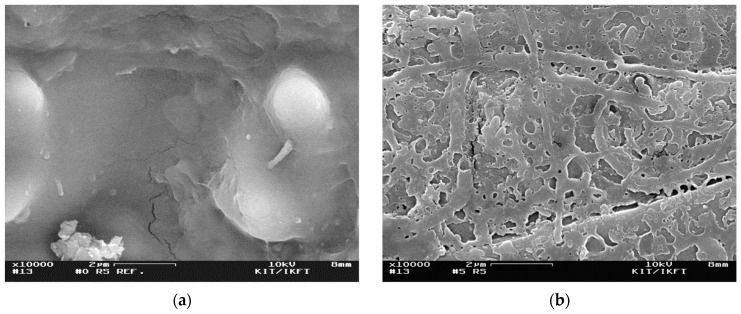

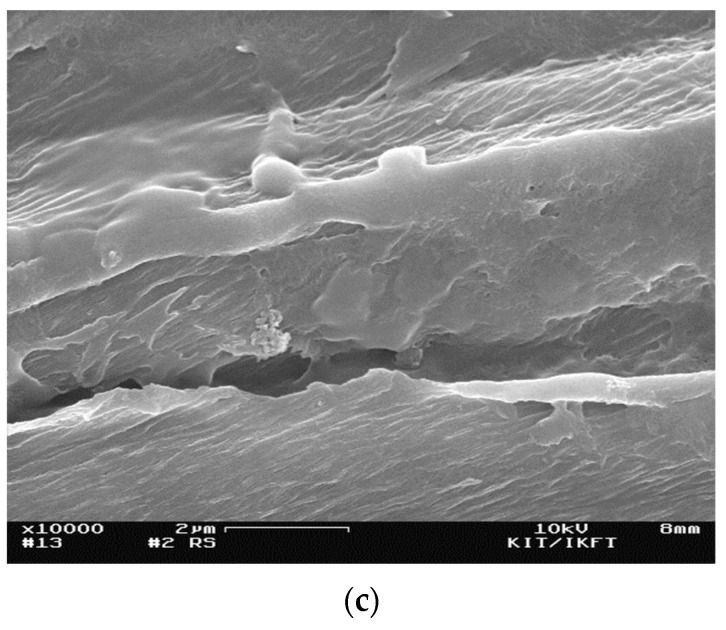
SEM images of (**a**) untreated rice straw and steam-exploded residue at a severity parameter of (**b**) *S_0_* = 4.10 min and (**c**) *S_0_* = 5.29 min.

The pretreated residue became darker and the particles more fragile with increasing severity. A difference in the biomass macrostructure was observed by SEM. After the steam explosion with a moderate severity parameter of *S_0_* = 4.10 min, a very porous structure was obtained (Figure 5b). However, at higher severities, no porous structure could be observed (Figure 5c). Photographs and SEM images of untreated rice straw and all steam-exploded residues are provided in Supplementary Materials. The surface structure for *S_0_* = 3.54 min indicated that parts of the solid melted and solidified later. Smaller fragments can be found, especially for high severities (e.g., *S_0_* = 4.20 min and *S_0_* = 4.48 min).

### 3.2. Specific Biogas Yields

The theoretical maximum gas production of the untreated rice straw was calculated according to Equation (3). The elemental composition measured of rice straw C_1.000_H_1.706_O_0.688_N_0.016_S_0.003_ resulted in a maximum methane yield of 0.478 m^3^_N_ kg _DM_^−1^.

**Figure 6:**
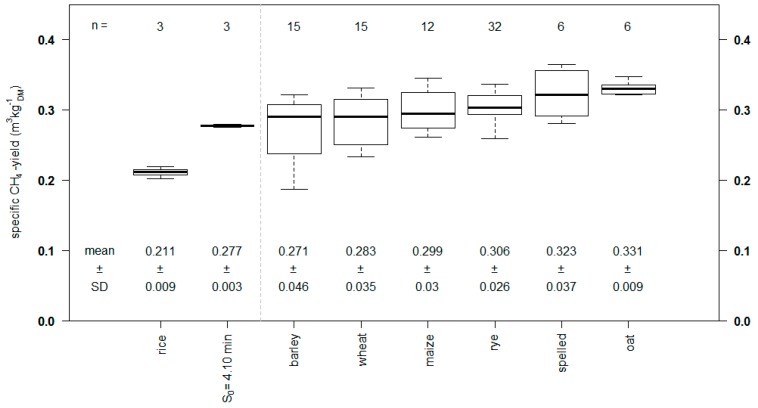
Specific methane yield of the untreated rice straw and the pretreated steam explosion variant *S_0_* = 4.10 min compared with other straws after 35 d (average yields of different samples regarding plant variety and particle size).

Figure 6 shows a comparison of the measured methane yield of rice straw with other common straws, such as wheat straw or maize straw, investigated in the Hohenheim biogas yield test. Rice straw showed a methane yield of 0.211 ± 0.009 m^3^_N_ kg _DM_^−1^, which was lower compared to other common straws. The steam explosion variant of rice straw with the highest increase in methane yield *S_0_* = 4.10 min reached the level of untreated wheat straw.

**Figure 7:**
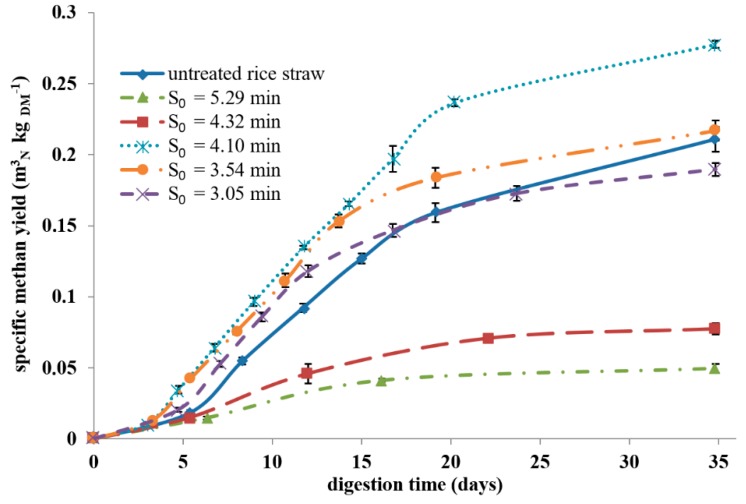
Cumulative specific methane production of rice straw and steam-exploded rice straw. Error bars indicate standard deviation for three repetitions.

Figure 7 shows the specific methane yield of the steam-exploded samples investigated over 35 d, which is also summarized in Table 3. The variants *S_0_* = 4.10 min and *S_0_* = 3.54 min reached a higher yield of 0.278 ± 0.003 m^3^_N_ kg _DM_^−1^ (+32%) and 0.217 ± 0.007 m^3^_N_ kg _DM_^−1^ (+3%) compared to untreated rice straw. These variants also showed faster gas production compared to the untreated straw. The other pretreatment conditions led to a reduction in specific methane yield of −76% (*S_0_* = 5.29 min), −63% (*S_0_* = 4.32 min), and −10% (*S_0_* = 3.05 min).

When steam explosion was performed at a low severity (*S_0_* = 3.05 min and *S_0_* = 3.54 min), the methane yield was similar to the untreated rice straw. Thus, we concluded that the pretreatment conditions were not severe enough to open up the structure of the lignocellulosic biomass for anaerobic digestion (see SEM images in Supplementary Materials). On the other hand, high-severity parameters (*S_0_* = 4.32 min and *S_0_* = 5.29 min) caused a drastic decrease in the methane yield. An obvious increase in the methane yield was obtained only when the steam explosion pretreatment was performed at moderate severities (*S_0_* = 4.10 min). At this severity, the particle morphology had the most porous structure among all conditions investigated (Figure 5b). Therefore, the broken macromolecular structure as well as additional surface had a positive effect on further degradation during anaerobic digestion. Thermogravimetric analysis showed a change of hemicellulose at *S_0_* = 4.10 min, but there was no formation of temperature-stable repolymerization products. The latter could be an explanation for the drastic decrease in biogas yields of the high-severity experiments. We assumed that repolymerization products from hemicelluloses were poorly digestible in the biogas process because of their high resistance against (1) hydrolytic cleavage (increased Klason lignin content, Figure 3b) and (2) decomposition reactions (increased thermal stability shown by decomposition at high temperatures of 380–480 °C, Figure 4).

## 4. Conclusions

Steam explosion of rice straw was performed as a pretreatment for anaerobic digestion to obtain a methane-rich biogas. Steam explosion was investigated at different reaction conditions, which resulted in severity parameters of *S_0_* = 3.05–5.29 min. The severity of steam explosion highly influences the methane yield:

- If the conditions of the steam explosion are too mild, the methane yield remains constant compared to untreated rice straw.
- If conditions are too severe, the methane yield drops dramatically. At these conditions, hemicelluloses are largely destroyed, and repolymerization leads to a more inert material.
- If conditions are moderate, the methane yield is increased, caused by a very porous structure and altered hemicellulose.

For process upscaling, the increase in methane yield at optimal steam explosion conditions has to be economically balanced with the mass loss of solid material during pretreatment and the effort of pretreatment itself.

## Figures and Tables

**Table 1:** Composition of the rice straw based on dry matter. (If standard deviation is indicated, four repetitions were performed.)

Ash Content after 550 °C	Ash Content after 1000 °C	Neutral Detergent Fiber	Acid Detergent Fiber	C	H	N	S	O	Si	K	Ca	Mg	Na
(wt.%)													
12.0 ± 0.1	10.7 ± 0.2	70.8 ± 2.8	46.2 ± 1.7	42.2	6.0	0.8	0.3	38.7	3.6	1.4	0.5	0.3	0.1

**Table 2:** Experimental setup for steam explosion of 27 g chipped rice straw.

Severity Parameter *S_0_* (min)	3.05	3.54	4.10	4.20	4.32	4.48	5.29
Steam input time (min)	30	30	30	16	12	30	32
Steam input mass (g)	150	150	150	78	60	150	60
Maximum reactor temperature (°C) ^1^	162	174	206	222	229	222	240
Maximum pressure (bar) ^1^	6.5	9.0	18.5	25.0	29.0	26.0	26.5

^1^ before explosion step (°C)

**Table 3:** Biogas yield, methane content, and specific methane yield of the untreated rice straw and steam-exploded rice straw after 35 d (mean with standard deviation of three repetitions).

Severity Parameter	Biogas Yield (m^3^_N_ kg _DM_^−1^)	CH_4_ (vol.%)	Specific Methane Yield (m^3^_N_ kg _DM_^−1^)
Untreated	0.368 ± 0.024	57.4 ± 1.5	0.211 ± 0.009
*S_0_* = 3.05 min	0.331 ± 0.009	57.3 ± 0.1	0.190 ± 0.005
*S_0_* = 3.54 min	0.393 ± 0.007	55.2 ± 0.9	0.217 ± 0.007
*S_0_* = 4.10 min	0.542 ± 0.011	51.3 ± 0.6	0.278 ± 0.003
*S_0_* = 4.32 min	0.128 ± 0.008	60.6 ± 2.0	0.078 ± 0.004
*S_0_* = 5.29 min	0.082 ± 0.002	60.8 ± 3.1	0.050 ± 0.003

## Supplementary Materials

The following are available online at https://www.mdpi.com/1420-3049/24/19/3492/s1, Figure S1. Temperature profile of steam explosion experiments that lead to different severity parameters *S_0_*; Table S1. Photographs and SEM images of untreated rice straw and steam-exploded residue.
