# Supplementary material for: Steam Explosion Conditions Highly Influence the Biogas Yield of Rice Straw
Source: Molecules. 2019 Sep 26;24(19):3492. doi: 10.3390/molecules24193492 (PMC6804039; doi:10.3390/molecules24193492)
Supplement: Supplementary file 1 [file molecules-24-03492-s001.pdf]

Supplementary materials

for the article

Steam explosion conditions influence the biogas yield of rice straw highly

David Steinbach, Dominik Wüst, Simon Zielonka, Johannes Krümpel, Simon Munder, Matthias Pagel and Andrea Kruse

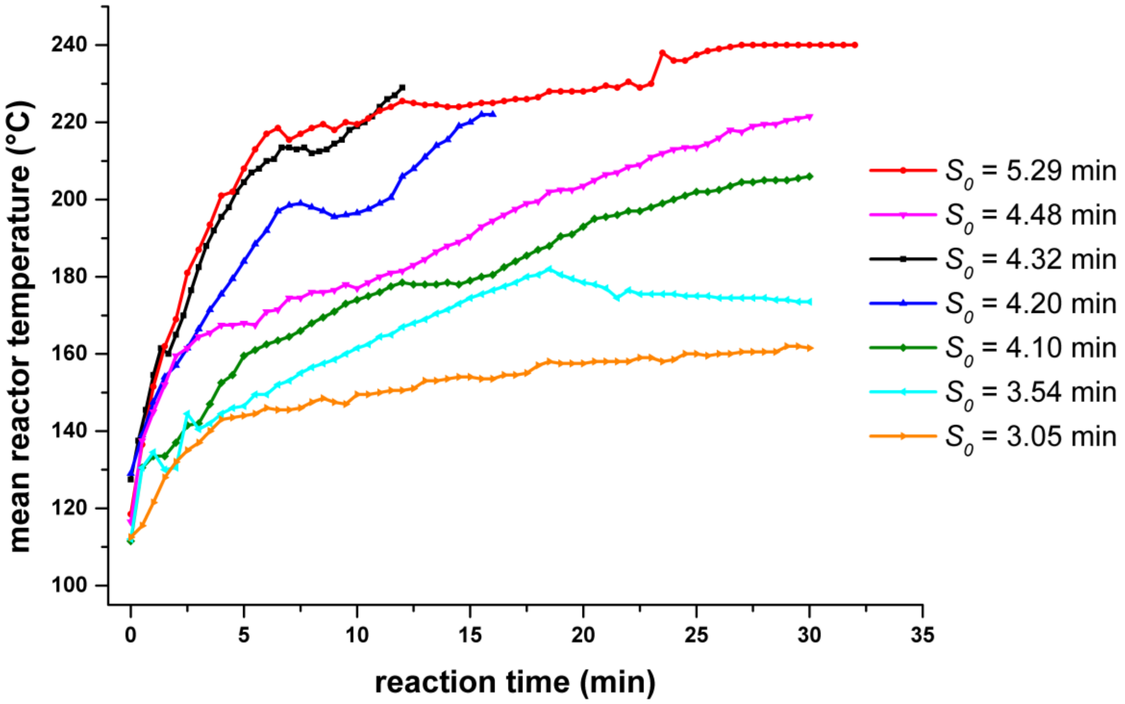

Figure S1. Temperature profile of steam explosion experiments which lead to different severity parameters S_0. A reaction time of zero is defined when the first steam enters the reactor. At the end of the temperature line the explosion step is performed.

Table S1. Photographs and SEM images of untreated rice straw and steam-exploded residue.

<table>
<tr><td>Untreated rice straw</td><td>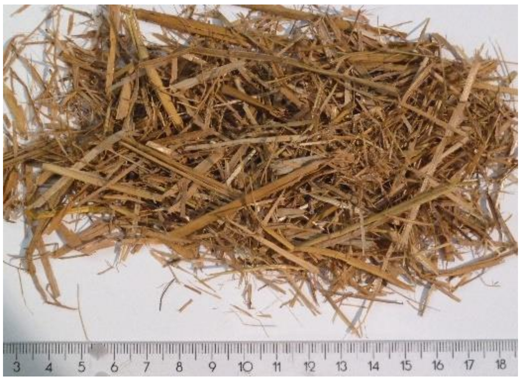</td><td>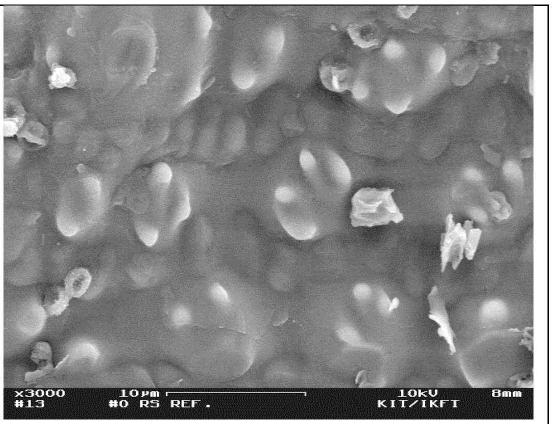
</td></tr>
<tr><td>S_0 = 3.05 min</td><td>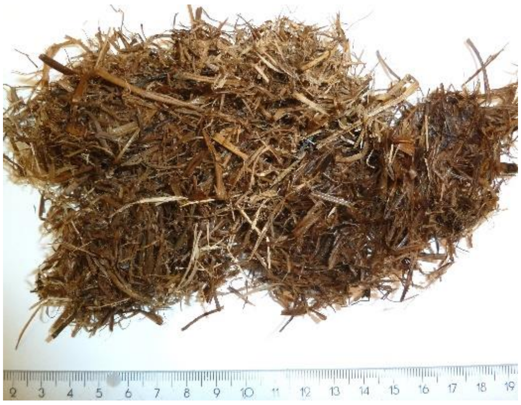</td><td>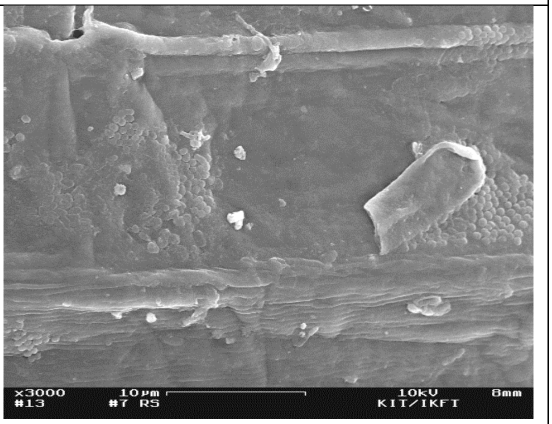
</td></tr>
<tr><td>S_0 = 3.54 min</td><td>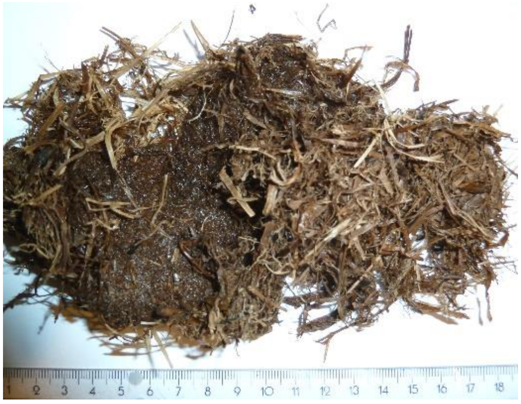</td><td>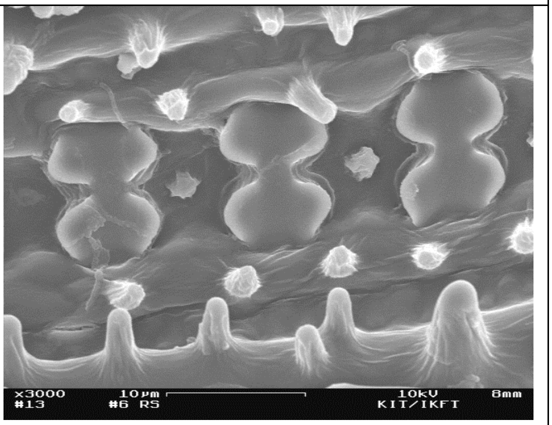
</td></tr>
<tr><td>S_0 = 4.10 min</td><td>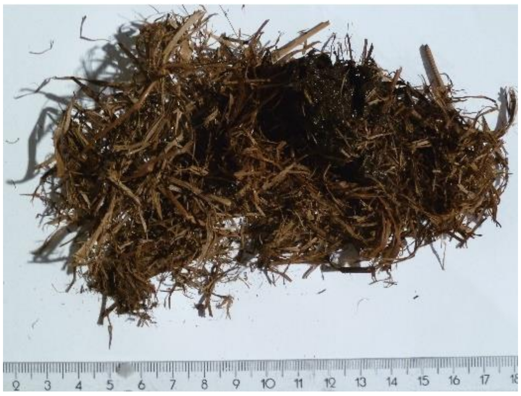</td><td>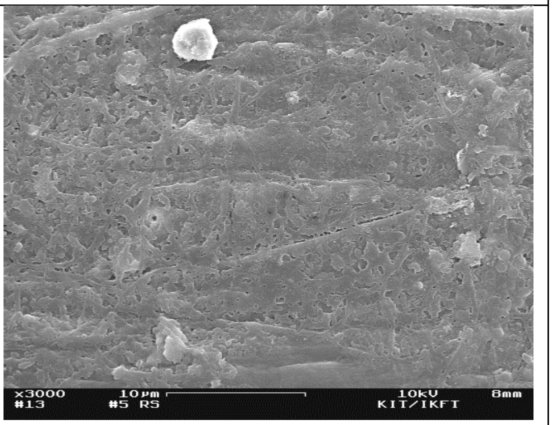
</td></tr>
</table>

S_0 = 4.20 min	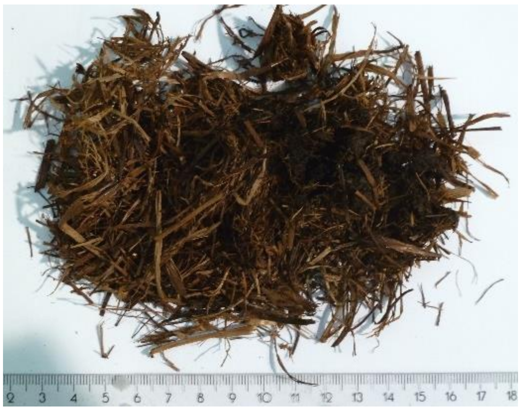	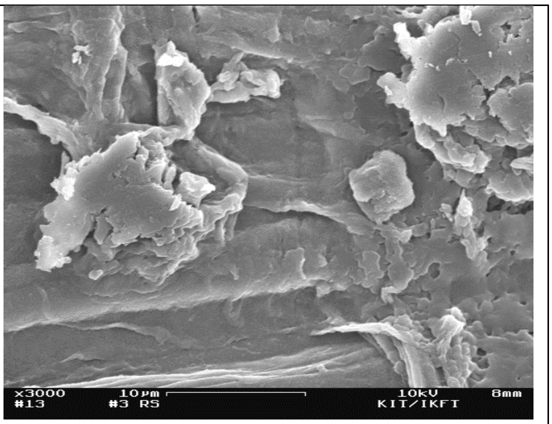
S_0 = 4.32 min	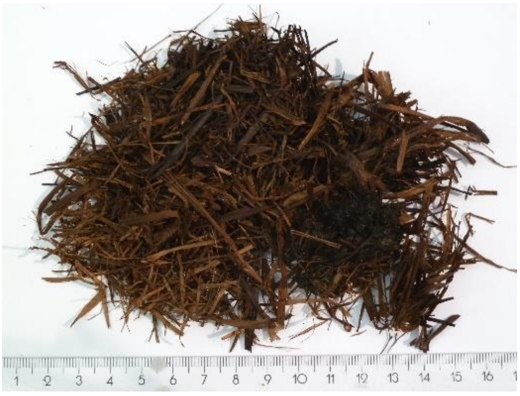	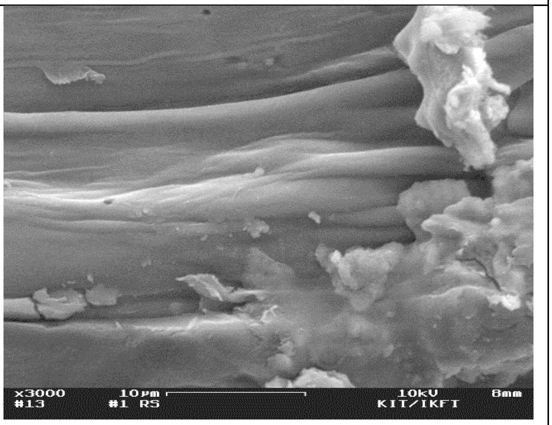
S_0 = 4.48 min	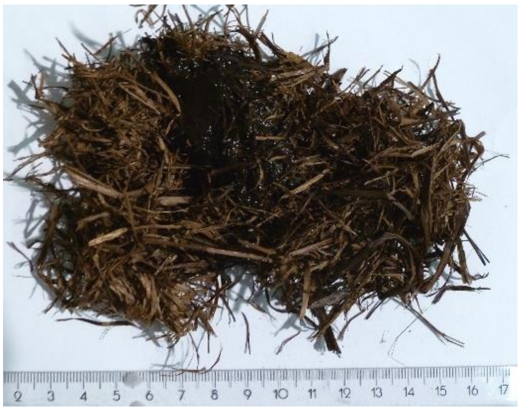	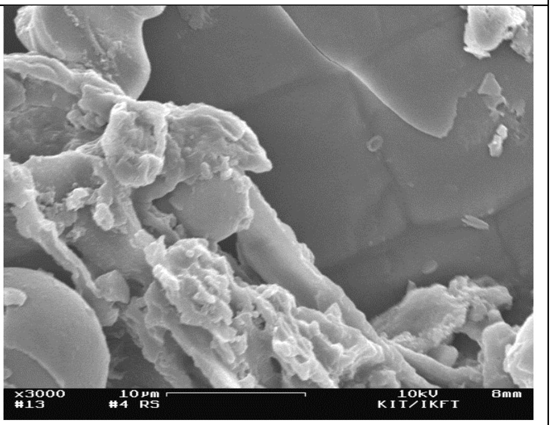
S_0 = 5.29 min	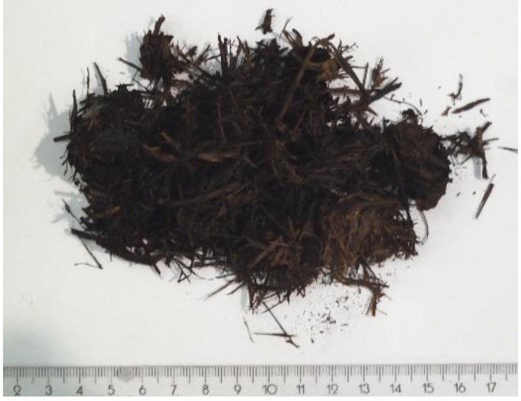	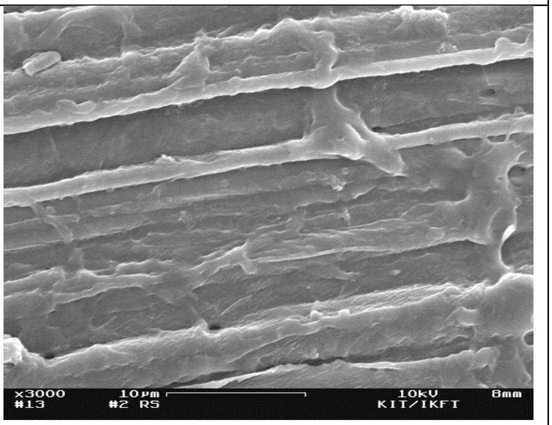
